# Integrative analysis of therapy resistance and transcriptomic profiling data in glioblastoma cells identifies sensitization vulnerabilities for combined modality radiochemotherapy

**DOI:** 10.1186/s13014-022-02052-z

**Published:** 2022-04-19

**Authors:** Leon Emanuel Schnöller, Valerie Albrecht, Nikko Brix, Alexander Edward Nieto, Daniel Felix Fleischmann, Maximilian Niyazi, Julia Hess, Claus Belka, Kristian Unger, Kirsten Lauber, Michael Orth

**Affiliations:** 1 Department of Radiation Oncology, University Hospital, LMU München, Marchioninistrasse 15, 81377 Munich, Germany; 2 German Cancer Consortium (DKTK), Munich, Germany; 3 German Cancer Research Center (DKFZ), Heidelberg, Germany; 4 Research Unit Radiation Cytogenetics, Helmholtz Center Munich, German Research Center for Environmental Health GmbH, Neuherberg, Germany; 5 Clinical Cooperation Group ‘Personalized Radiotherapy in Head and Neck Cancer’ Helmholtz Center Munich, German Research Center for Environmental Health GmbH, Neuherberg, Germany

**Keywords:** Glioblastoma, Radiotherapy, Temozolomide, Therapy resistance, Radiosensitization, Chemosensitization, Clonogenic survival, Correlation analysis, DNA damage response, ATR, LIG4, ATM

## Abstract

**Background:**

Inherent resistance to radio/chemotherapy is one of the major reasons for early recurrence, treatment failure, and dismal prognosis of glioblastoma. Thus, the identification of resistance driving regulators as prognostic and/or predictive markers as well as potential vulnerabilities for combined modality treatment approaches is of pivotal importance.

**Methods:**

We performed an integrative analysis of treatment resistance and DNA damage response regulator expression in a panel of human glioblastoma cell lines. mRNA expression levels of 38 DNA damage response regulators were analyzed by qRT-PCR. Inherent resistance to radiotherapy (single-shot and fractionated mode) and/or temozolomide treatment was assessed by clonogenic survival assays. Resistance scores were extracted by dimensionality reduction and subjected to correlation analyses with the mRNA expression data. Top-hit candidates with positive correlation coefficients were validated by pharmacological inhibition in clonogenic survival assays and DNA repair analyses via residual γH2AX/53BP1-foci staining.

**Results:**

Inherent resistance to single-shot and similarly also to fractionated radiotherapy showed strong positive correlations with mRNA expression levels of known vulnerabilities of GBM, including PARP1, NBN, and BLM, as well as ATR and LIG4—two so far underestimated targets. Inhibition of ATR by AZD-6738 resulted in robust and dose-dependent radiosensitization of glioblastoma cells, whereas LIG4 inhibition by L189 had no noticeable impact. Resistance against temozolomide showed strong positive correlation with mRNA expression levels of MGMT as to be expected. Interestingly, it also correlated with mRNA expression levels of ATM, suggesting a potential role of ATM in the context of temozolomide resistance in glioblastoma cells. ATM inhibition exhibited slight sensitization effects towards temozolomide treatment in MGMT low expressing glioblastoma cells, thus encouraging further characterization.

**Conclusions:**

Here, we describe a systematic approach integrating clonogenic survival data with mRNA expression data of DNA damage response regulators in human glioblastoma cell lines to identify markers of inherent therapy resistance and potential vulnerabilities for targeted sensitization. Our results provide proof-of-concept for the feasibility of this approach, including its limitations. We consider this strategy to be adaptable to other cancer entities as well as other molecular data qualities, and its upscaling potential in terms of model systems and observational data levels deserves further investigation.

## Background

Radio/chemotherapy is a key treatment option for glioblastoma (GBM), the most common and most aggressive type of primary brain tumor [1–5]. However, GBM is known for its high levels of inherent treatment resistance, giving rise to early recurrence and dismal prognosis with a median survival time of less than 15 months [2, 6, 7]. For patients with recurrent GBM, treatment perspectives are even worse, because therapy resistance has manifested and/or emerged in the course of primary treatment [8, 9]. In order to increase the efficacy of radio/chemotherapy, it is inevitable to identify regulators that drive radio/chemoresistance as potential vulnerabilities for molecularly targeted radio/chemosensitization strategies in combined modality treatment approaches [10, 11].

The DNA damage response (DDR), a conserved network of signaling and DNA repair pathways [12], is of particular interest in this context, since both the expression and the mutational status of DDR genes are frequently altered in malignant cells—especially in GBM [13–15]. Mechanistically, increased and/or accelerated DNA damage repair capacity, enhanced detoxification of reactive oxygen species (ROS), and deficiencies in cell death regulation are frequently involved [16, 17].

Using GBM as a paradigm for a highly treatment-resistant cancer, we developed a two-step integrative approach to identify genes within the DDR network whose expression levels are associated with the degree of radio/chemoresistance in cancer cells. We performed transcriptomic profiling of 38 DDR genes in a panel of seven human GBM cell lines and correlated their expression levels with scores of inherent therapy resistance against ionizing radiation (IR) or temozolomide (TMZ) treatment as extracted by dimensionality reduction of clonogenic survival data. This approach allowed us to identify several DDR genes whose mRNA expression levels showed strong positive correlations with inherent treatment resistance in GBM cells. The druggable top hits were chosen for evaluation in targeted radio/chemosensitization experiments. Proof-of-concept for our approach was provided along several lines, including the confirmation of MGMT mRNA expression as top correlating feature for TMZ resistance and the identification of ATR expression as top correlating feature for radioresistance which was validated by significant radiosensitization of GBM cells upon ATR inhibition in vitro. Nevertheless, not all correlating features could be validated by pharmacological perturbation. Hence, our study provides a blueprint for systematic screens of radio/chemotherapy resistance in preclinical oncology which is evidently transferable to other cancer entities and has promising upscaling potential with regard to the observational data levels.

## Methods

### Cell lines and reagents

The human GBM cell lines A172, LN18, LN229, and T98G were purchased from American Type Culture Collection (ATCC, Manassas, VA, USA), and the human GBM cell lines U87-MG, U138-MG, and U251-MG were purchased from Cell Lines Service GmbH (CLS, Eppelheim, Germany). All cell lines were cultured in Dulbecco's Modified Eagle Medium (DMEM) supplemented with 10% (v/v) heat-inactivated fetal calf serum (FCS), 100 U/ml penicillin, and 0.1 mg/mL streptomycin (all from ThermoScientific, Schwerte, Germany) at 37 °C and 7.5% CO_2_. The cell lines were maintained at low passage numbers (< 10 passages) and were regularly tested to be free from mycoplasma (MycoAlert, Lonza; Basel, Switzerland). Cell line identity was confirmed by short tandem repeat (STR) typing (service provided by the DSMZ, Braunschweig, Germany).

The ATR inhibitor AZD-6738 and the ATM inhibitor KU-60019 were both purchased from Absource GmbH (Munich, Germany) and dissolved at 10 mM in dimethylsulfoxide (DMSO, Sigma-Aldrich, Taufkirchen, Germany). The LIG4 inhibitor L189 was purchased from Bio-Techne GmbH (Wiesbaden-Nordenstedt, Germany), and dissolved at 100 mM in DMSO. Temozolomide (TMZ) was purchased from Sigma-Aldrich and dissolved at 100 mM in DMSO. Final concentrations of all drugs tested in the assays were adjusted by directly diluting the respective stock solutions in cell culture medium. Appropriate vehicle dilutions served as controls.

### X-ray treatment

Irradiation of cells was performed with an RS-225 X-ray cabinet (X-strahl, Camberley, Great Britain) at 200 kV and 10 mA (Thoraeus filter, 1 Gy in 243 s) as described [18].

### Quantitative real-time PCR (qRT-PCR)

Quantification of mRNA expression levels of DNA damage response (DDR) genes was performed by quantitative real-time RT-PCR (qRT-PCR) as previously described [18–20]. Briefly, total RNA was extracted from cells using the NucleoSpin RNA II extraction kit (Macherey & Nagel, Dueren, Germany). 0.5–1 µg of isolated RNA were mixed with 10 U/µl RevertAid transcriptase, 5 µM Oligo(dT)_18_, 5 µM random hexamers, 1 U/µl Ribolock RNase inhibitor (all from Thermo Scientific), and 500 µM dNTPs (Promega, Heidelberg, Germany) and reversely transcribed. Subsequently, 4 or 20 ng of cDNA were mixed with 300 nM forward and reverse primers in 1× Maxima SYBR Green qPCR Master Mix (ThermoScientific), and subjected to qRT-PCR. qRT-PCR runs were performed with a standard cycling protocol (10 min 95 °C, 45× (15 s 95 °C, 30 s 60 °C)) on an LC480 qPCR cycling platform (Roche Applied Science, Penzberg, Germany). Relative quantification was performed using the ddCT method. Efficiency correction was implemented by using the two different cDNA concentrations (4 and 20 ng cDNA per reaction). Results were normalized to a matrix comprised of three reference genes (18S rRNA, 5’-Aminolevulinate Synthase-1 (ALAS), and β2-Microglobulin (B2M)), and calibrated to the relative expression levels measured for human astrocytes (BioCat, Heidelberg, Germany). Three replicates were analyzed per cell line. Primer sequences have been previously described [19, 20]. Expression values of LN229 and T98G cells had been published before [18] and were re-analyzed for this study.

### Determination of MGMT promoter methylation in methylome array

For array-based methylome analysis, DNA was extracted from cells by Qiagen Allprep DNA/RNA mini kit (Qiagen, Hilden, Germany). 500 ng of genomic DNA were subjected to hybridization on Infinium EPIC methylation arrays (Illumina, San Diego, CA; USA), and analyzed according to the manufacturer's instructions. Assays were scanned, and idat files were imported in R using the minfi package and processed according to the Illumina BeadStudio workflow.

### Clonogenic survival assay

Clonogenic survival was determined by colony formation assays as described [21, 22]. Briefly, cells were detached with Trypsin/EDTA (ThermoScientific), counted with a Neubauer counting chamber, and up to 55,000 cells per well were seeded into 6-well plates yielding a range of 10–200 colonies after treatment. Cell adherence was allowed for 4 h. For radiation experiments, medium was replaced, cells were irradiated once (single-shot mode) or every 24 h (fractionated mode) with the indicated doses, and incubated at 37 °C and 7.5% CO_2_ for up to 12 d to permit colony formation. In case of TMZ treatment, cells were incubated with the indicated TMZ concentrations for 24 h, medium was replaced by TMZ-free medium, and colony formation was allowed for up to 12 d. For combined treatment, cells were additionally irradiated with 0–10 Gy before incubation. For targeted sensitization experiments, the inhibitors were added at the indicated concentrations 30–60 min before IR or TMZ treatment. 24 h later, medium was replaced by inhibitor-free medium.

Colonies were fixed in 80% ethanol and stained with 0.8% methylene blue (both from Merck Millipore, Darmstadt, Germany). All colonies that contained at least 50 cells were counted with a Stemi 305 stereomicroscope (Carl Zeiss, Oberkochen, Germany). Percentages of colony forming cells were calculated and normalized to the respective plating efficiencies at approximately matched colony numbers. Survival curves were subjected to linear-quadratic (for radiation experiments) or logistic (for TMZ treatment) fitting, respectively. At least three independent experiments were performed.

### SDS-PAGE and Western Blot

Reducing gradient SDS-PAGE and western blot analyses were performed as previously described [18]. Briefly, cells from cryostocks were thawed, washed with PBS, and lysed with lysis buffer (50 mM Tris–HCl pH 7.6, 150 mM NaCl, 1% Triton X-100 (v/v, all from Sigma-Aldrich), 1 × EDTA-free protease inhibitor cocktail (Roche)) for 30 min on ice. Protein concentrations were measured by Bradford assay (BioRad, Feldkirchen, Germany). 20 or 400 µg of total protein were subjected to gradient (6–15%) SDS-PAGE, and proteins were transferred onto PVDF Immobilon FL membranes (Merck Millipore). Membranes were blocked with 5% low-fat milk powder (Carl Roth, Karlsruhe, Germany) dissolved in TBST buffer (13 mM Tris–HCl pH 7.5, 150 mM NaCl, 0.02% Triton X-100 (v/v)), and incubated with primary antibodies detecting MGMT (Biozol, Eching, Germany) or vinculin (Sigma-Aldrich) at 4 °C overnight. After washing with TBST, membranes were incubated with IRDye800-conjugated secondary antibodies (LI-COR Biosciences, Bad Homburg, Germany) for 1 h at room temperature. Upon additional washing with TBST, IR800 dye fluorescence was measured with an Odyssey scanner (LI-COR Biosciences).

### Immunofluorescence microscopy

Immunofluorescence staining of phosphorylated histone variant H2AX (γH2AX) and p53 binding protein 1 (53BP1) was performed as described [18] and was used to analyze the efficacy of DNA damage repair upon inhibition of identified candidates in terms of residual γH2AX/53BP1-positive DNA damage repair foci 20 h after irradiation. Briefly, 20,000 cells were seeded into 24-well plates supplemented with coverslips and allowed to adhere overnight. Cells were treated with 1.0 μM AZD-6738, 50 μM L189, or DMSO (control) for 1 h before being irradiated at 4 Gy. Cells were fixed 20 h after IR with 3.7% isotonic paraformaldehyde (Merck Millipore) containing 0.1% Triton X-100 (v/v, Sigma-Aldrich) and permeabilized with 0.5% isotonic Triton X-100 (v/v). Unspecific antibody binding was blocked by incubation in 3% isotonic bovine serum albumin (BSA, w/v, Sigma-Aldrich) containing 0.1% Triton X-100 (v/v) for 1 h at room temperature. Cells were stained with monoclonal mouse anti-γH2AX antibody (S139, Merck Millipore) and polyclonal rabbit anti-53BP1 antibody (Bio-Techne), both diluted in 3% isotonic BSA, for 1 h at room temperature. After washing with PBS containing 0.1% Triton X-100 (v/v), cells were stained for 1 h with AlexaFluor488-coupled goat-anti-mouse IgG and AlexaFluor568-coupled goat-anti-rabbit IgG (both from ThermoScientific). DNA was stained with Hoechst 33342 (2.0 µg/ml, Sigma-Aldrich). Upon washing with PBS (0.1% Triton X-100 (v/v)), coverslips were mounted with 4 µl Fluoromount mounting medium (Sigma-Aldrich) onto microscope slides. Microscopic analysis was performed with a Zeiss AxioObserver Z1 inverted microscope equipped with an AxioCam MR Rev3 camera, an LCI Plan-Neofluar 63x/1.3 glycerol objective, and ZEN 2.3 software (all from Carl Zeiss, Oberkochen, Germany). For image acquisition, 31 z-stacks with 250 nm interstack distance were captured, and image deconvolution was performed with the ZEN 2.3 software.

### Statistical analysis

Statistical analyses, if not stated otherwise, were performed with OriginPro (OriginLab Ltd., Northhampton, MA, USA). Results are presented as individual data points from individual biological replicates. Group comparisons were performed by two-way ANOVA upon confirmation of normal distribution. Unsupervised hierarchical clustering and principal component analysis (PCA) of z-transformed data were performed as described [19, 21].

## Results

### mRNA expression levels of DNA damage response (DDR) regulator genes are frequently altered in human GBM cells

**Fig. 1:**
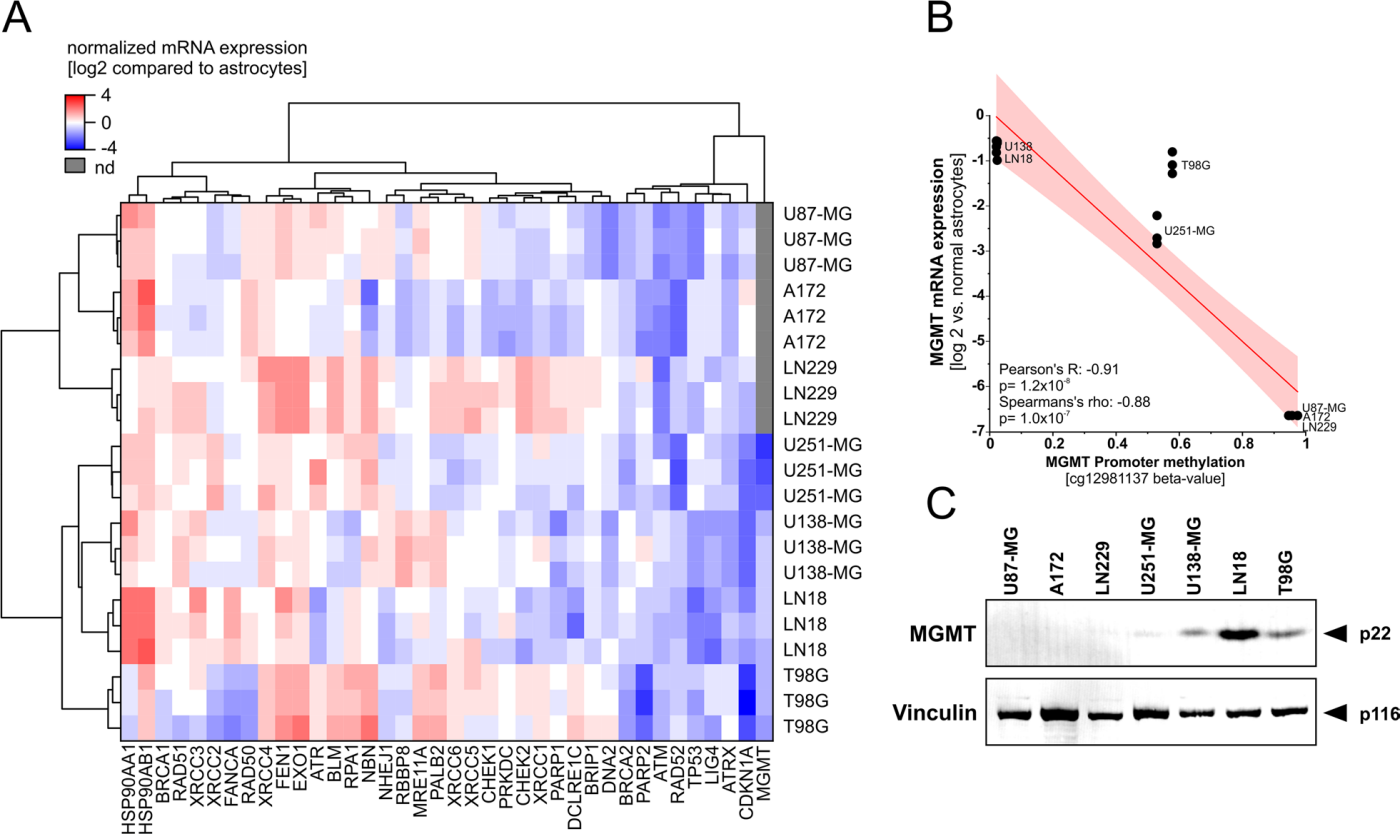
mRNA expression analysis of DNA damage response (DDR) regulator genes, and MGMT promoter methylation analysis in human GBM cell lines. **A** Basal mRNA expression levels of DDR regulator genes in GBM cell lines A172, LN18, LN229, T98G, U87-MG, U138-MG, and U251-MG as measured by qRT-PCR (ddCT method). Expression levels were normalized to a matrix of three reference genes (18S rRNA, β2-Microglobulin, and 5’-Aminolevulinate Synthase-1), and calibrated on the expression levels of astrocytes. Three replicates were analyzed per cell line. Expression values (log2-transformed) and samples were subjected to unsupervised hierarchical clustering. **B** Correlation of MGMT promoter methylation status and MGMT mRNA expression levels in the employed GBM cell lines as detected by methylome array and qRT-PCR, respectively. **C** Protein expression levels of MGMT in GBM cells as measured by western blot. Vinculin served as loading control

The predominant effect of ionizing radiation (IR) and DNA-targeted chemotherapy on cancer cells is the induction of DNA damage of which DNA double-strand breaks are most severe, ultimately leading to cell cycle arrest, cell death, and abrogation of clonogenic survival [23]. Accordingly, resistance against DNA damage-induced cell death and clonogenic inactivation is a major obstacle in current GBM treatment [24], and frequently derives from alterations both in abundance and functionality of DNA damage response (DDR) regulators [25]. In the present study, we examined the basal mRNA expression levels of 38 DDR genes in a panel of seven human GBM cell lines in comparison to primary human astrocytes and observed a thorough and massive upregulation of multiple DDR genes analyzed (Fig. 1A). This is in line with previous reports showing that the abundance of DDR proteins and DDR function are increased in GBM cells [18, 26, 27]. Several of the genes found to be upregulated in our study have been reported to be involved in the recognition of DNA damage and upstream DDR signaling, such as the proximal DDR kinase Ataxia Telangiectasia and RAD3 Related (ATR), the MRN complex components Meiotic Recombination 11 Homolog A (MRE11A), RAD50, and Nibrin (NBN) [28], or the single strand DNA-binding Replication Protein A1 (RPA1), respectively. Other genes found to be upregulated included Flap-structure-specific Endonuclease 1 (FEN1), Exonuclease 1 (EXO1), Heat Shock Proteins 90AA1 and 90AB1 (HSP90AA1 and HSP90AB1), and members of the X-ray Repair Cross-Complementing (XRCC) family.

We also detected some genes whose mRNA expression levels were downregulated in GBM cells as compared to normal healthy astrocytes (Fig. 1A). Among these genes, several candidates with documented tumor-suppressive functions were found, such as TP53, and CDKN1A [29, 30]. Interestingly also, the gene encoding for O^6^-Methylguanine-DNA-methyltransferase (MGMT), a DNA repair enzyme known to remove alkylating modifications from DNA thereby counteracting the anti-tumorigenic effect of temozolomide (TMZ) [31], was in principal downregulated in GBM cells as compared to normal astrocytes, and in several GBM cell lines it was not detectable at all, both on mRNA and protein level (Fig. 1A and C).

To analyze this aspect in further detail, we also determined the methylation status of the MGMT promoter in our GBM cell line panel by methylome arrays (Fig. 1B). As expected, MGMT promoter methylation and mRNA expression levels showed an inverse relation, and the mRNA expression levels were in good accordance with the observed protein expression levels (Fig. 1A and B). In summary, our DDR transcriptomic profiling screen revealed that basal mRNA expression levels of DDR genes are frequently altered in human GBM cells, suggesting potential vulnerabilities for targeted inhibition and sensitization strategies in combined modality treatment approaches.

### Human GBM cell lines exhibit different levels of inherent resistance towards IR, TMZ or the combination thereof in clonogenic survival assays

**Fig. 2:**
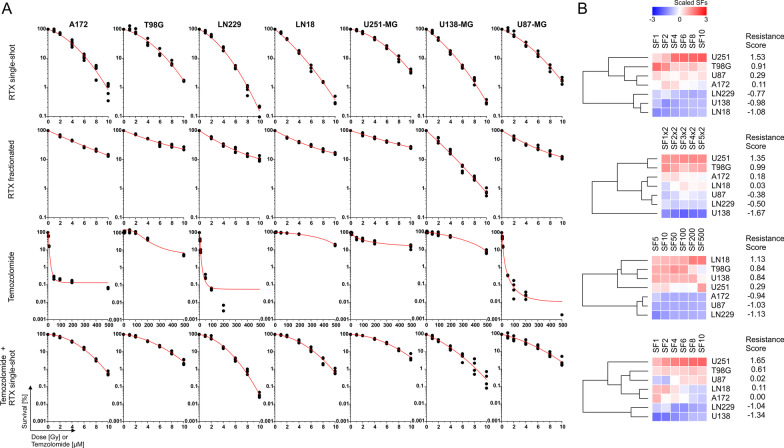
Clonogenic survival of GBM cells upon treatment with X-ray IR, TMZ, and the combination thereof. **A** Clonogenic survival of A172, T98G, LN229, LN18, U251-MG, M138-MG, and U87-MG cells after single-shot IR (0–10 Gy), fractionated IR (0–5 fractions of 2 Gy each), TMZ treatment (0–500 μM for 24 h), and combination of 5 µM TMZ (24 h) and single-shot IR (0–10 Gy) as determined by colony formation assays. Results of at least three independent experiments are depicted. Super-imposed fitting functions are linear-quadratic in case of radiation experiments and logistic in case of TMZ treatment. **B** Z-transformed survival data are depicted as unsupervised hierarchical clustering. Principal component analysis (PCA)-derived resistance scores (scores of PC1 as described in [21]) are shown

To identify differences in inherent resistance towards IR and TMZ across our GBM cell line panel, we performed clonogenic survival assays. In analogy to our previous studies with other cancer entities, clonogenic survival data were subjected to dimensionality reduction via principal component analysis (PCA), and the extracted scores of the first principal component (PC1) were used as a measure of treatment resistance [19–21] (Fig. 2). We observed marked differences in clonogenic survival after single-shot IR as well as after fractionated IR (5 × 2 Gy). U251-MG, T98G, and U87-MG revealed highest levels of inherent resistance to single-shot IR, while LN18, U138-MG, and LN229 were most sensitive. Similarly, resistance to fractionated IR was highest in U251-MG, and T98G cells, while U138-MG and LN229 again showed highest sensitivity. Resistance to TMZ treatment, on the contrary, was highest in LN18, T98G, and U138-MG cells, the cell lines with the lowest levels of MGMT promoter methylation (Fig. 1B) and highest levels of MGMT mRNA expression (Fig. 1A). The combined treatment comprising single-shot IR and TMZ displayed a similar resistance pattern as the single-shot IR only treatment (Fig. 2). Again, highest levels of resistance were observed in U251-MG, T98G, and U87-MG cells, while U138-MG, and LN229 were most sensitive. However, the additive effect of both treatment modalities was clearly observed exemplarily at the LN18 cell line which showed highest sensitivity to single-shot IR mono-treatment, and highest resistance to TMZ mono-treatment, resulting in an intermediate resistance score for the combination treatment.

### Correlation of therapy resistance data with transcriptomic profiling data identifies ATR, LIG4, and ATM as potential candidates for targeted sensitization of GBM cells

**Fig. 3:**
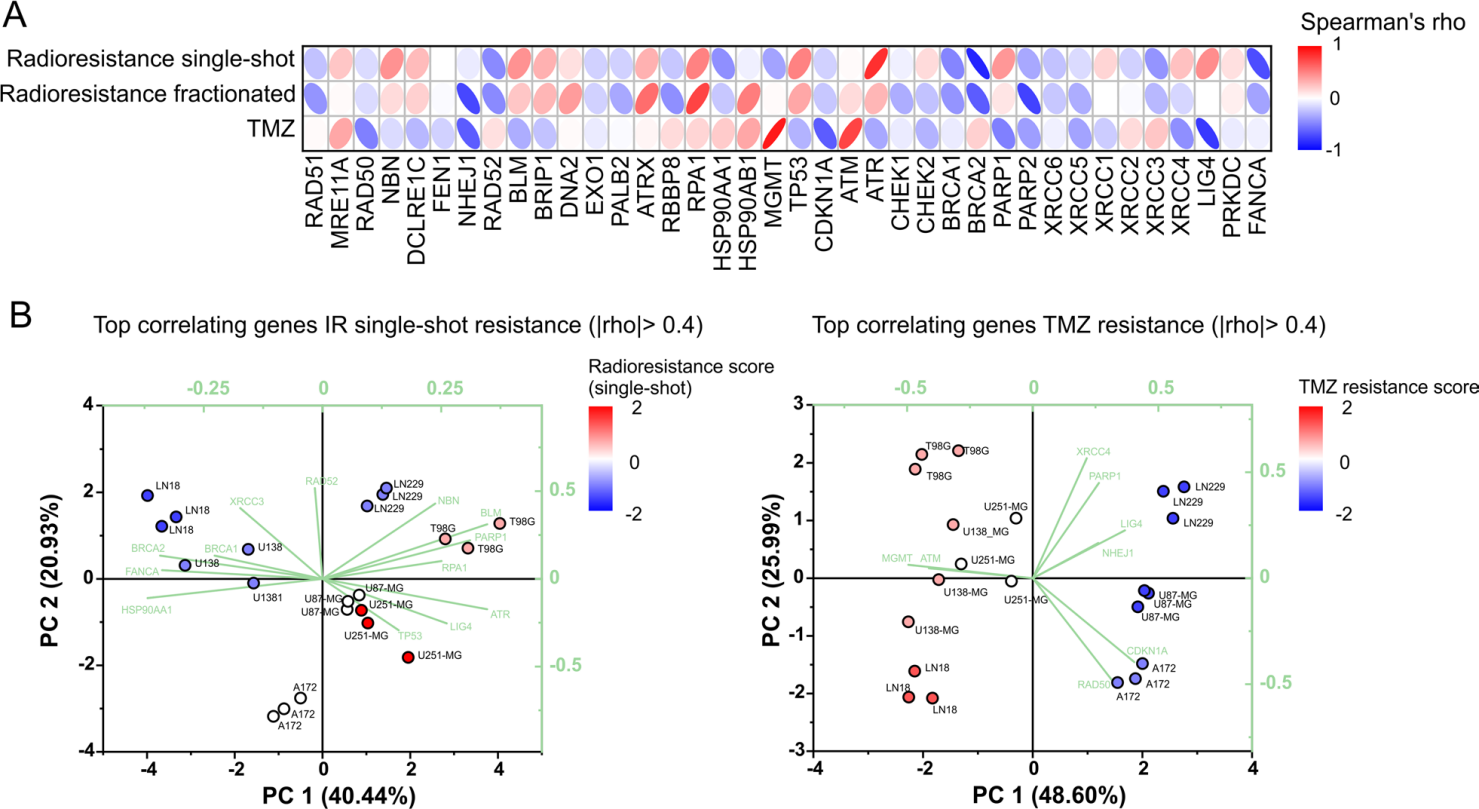
Correlation analysis of DDR mRNA expression levels with inherent treatment resistance scores. **A** Spearman's correlation analysis of DDR regulator mRNA expression levels (Fig. 1) with scores of inherent resistance (scores of PC1) to single-shot IR, fractionated IR, and TMZ treatment (Fig. 2). **B** PCA-Biplot of genes with |rho|> 0.4 for single-shot IR (left panel) and TMZ treatment (right panel)

In order to identify markers and potential drivers of inherent resistance against IR and TMZ in GBM cells, we combined the data from our qRT-PCR and radio/chemoresistance screens (Figs. 1A and 2) and performed Spearman correlation analyses of the therapy resistance scores (PC1s) with the mRNA expression levels of the 38 DDR genes (Fig. 3). For IR applied in single-shot mode, we observed the highest positive correlation for ATR, suggesting an involvement of ATR in GBM radioresistance. We also obtained positive correlations for TP53, RPA1, LIG4, NBN, BLM, and PARP1 offering interesting opportunities for molecularly targeted radiosensitization approaches. Highly sophisticated inhibitors are available for some of these candidates, including ATR and PARP1 [32–34]. For LIG4, first-line inhibitors, such as L189, have recently been developed [35], and the MRN complex comprising NBN, and its satellite BLM has been shown to be vulnerable for instance to inhibition of HSP90 chaperoning activity [36]. The radiosensitizing effects of PARP1 and HSP90 inhibition on GBM cells have been shown by many groups including ours [18, 37–39], thus providing principal validating evidence for our correlation approach and confirming the functional involvement of the potential target genes identified. ATR and LIG4 were picked as novel targets in GBM, and so we decided to examine the effects of ATR and LIG4 inhibition on radiosensitization of GBM cells in greater detail. Correlation of the fractionated radioresistance scores with the transcriptomic data revealed in principal similar trends, yet with slightly different ranking. Here, RPA1, ATRX, and HSP90AB1 were the top positively correlating hits (Fig. 3).

Resistance against TMZ showed the highest positive correlation with the expression levels of MGMT, once more confirming the feasibility of our methodological approach (Fig. 3). However, it also showed a strong positive correlation with expression of ATM, the major upstream signaling kinase of the DDR network [40] (Fig. 3). This finding was rather unexpected, since it suggested that ATM might play a hitherto unknown role in TMZ resistance of GBM cells. Therefore, we decided to test this hypothesis by interfering with ATM kinase activity in combination with TMZ treatment [41].

### Functional validation confirms inhibition of ATR but not LIG4 as a strategy to increase the efficacy of radiotherapy in GBM

We proceeded to validate our candidates by pharmacological interference with their functions. Deliberately, we concentrated on ATR and LIG4 in terms of sensitization towards single-shot IR and on ATM in terms of sensitization towards TMZ treatment. The other candidates were left out either because of their undruggability (e.g. RPA1, ATRX), or because of already existing evidence for their performance as sensitization targets in GBM (PARP1 and MRN complex) [36–38, 42, 43].

**Fig. 4:**
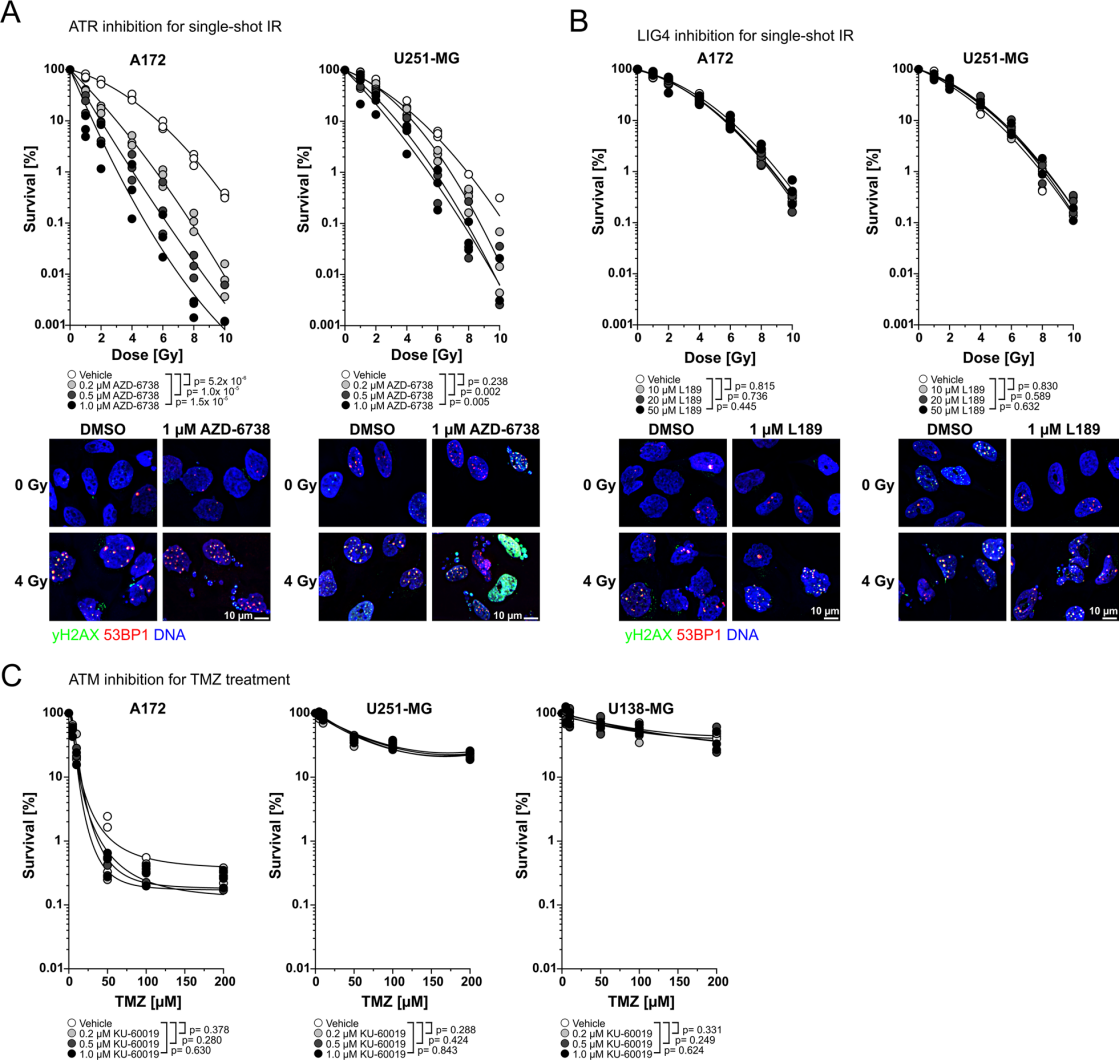
Validation of candidate genes by targeted inhibition and clonogenic survival assays. **A** Clonogenic survival of A172 and U251-MG cells after single-shot IR (0–10 Gy) and ATR inhibition by AZD-6738 (0–1.0 μM, upper panel). Immunofluorescence staining of γH2AX and 53BP1 20 h after irradiation at 4 Gy ± ATR inhibition by 1 µM AZD-6738 (lower panel). γH2AX is depicted in green, 53BP1 in red, and DNA is depicted in blue. Scale bar represents 10 µm. **B** Clonogenic survival of A172 and U251-MG cells after single-shot IR and LIG4 inhibition by L189 (0–50 μM, upper panel). Immunofluorescence staining of γH2AX and 53BP1 20 h after irradiation at 4 Gy ± LIG4 inhibition by 50 µM L189 as performed in A (lower panel). **C** Clonogenic survival of A172, U138-MG, and U251-MG cells after 24 h treatment with TMZ (0–200 μM) and ATM inhibition by KU-60019 (0–1.0 μM). Super-imposed fitting functions are linear-quadratic in case of radiation experiments and logistic in case of TMZ treatment. Results of three independent experiments are depicted for each condition, and p-values were calculated by two-way ANOVAs

For ATR inhibition, we made use of the highly brain-penetrable ATR inhibitor AZD-6738 [44, 45]. As cell models, one GBM cell line of moderate radioresistance (A172) and one cell line of high radioresistance (U251-MG) were chosen (Fig. 2). Inhibitor treatment was performed for 24 h, starting 1 h before irradiation. Afterwards, medium was replaced and colony formation was allowed in inhibitor-free medium. Both cell lines were convincingly sensitized to single-shot IR treatment by AZD-6738 in a dose-dependent manner (Fig. 4A), confirming that ATR indeed could serve as a target for radiosensitization in GBM [46]. This was also supported by analyses of residual DNA damage upon irradiation as detected by immunofluorescence staining of phosphorylated histone variant H2AX (γH2AX) and 53BP1, two well-established markers of DNA damage repair foci [47, 48]. We observed an accumulation of residual γH2AX/53BP1-foci 20 h after single-shot irradiation at 4 Gy in the presence of AZD-6738. Additionally, the combination treatment resulted in a massive increase in micronuclei formation in both cell lines analyzed (Fig. 4A), indicating a reduction in DNA damage repair efficacy in GBM cells upon ATR inhibition which should be further investigated in in vivo models. We next interrogated whether inhibition of LIG4 could also sensitize GBM cells to IR. LIG4 is the major DNA ligase of the non-homologous end joining (NHEJ) pathway [49], rendering it a promising target for sensitization attempts. Inhibition experiments were performed in analogy to the procedure described for ATR inhibition. However, inhibition of LIG4 activity by the poly-DNA-ligase inhibitor L189 [35] exhibited no obvious effects on radioresistance of GBM cells (Fig. 4B), at least in the cell lines tested (A172, U251-MG). We also did not observe marked differences in residual γH2AX/53BP1 repair foci, nor in the formation of micronuclei (Fig. 4B), again confirming that L189 had no obvious effects on the response of A172 and U251-MG cells to ionizing irradiation. Technically, we cannot rule out that this derives from the suboptimal affinity and/or specificity of the L189 inhibitor for LIG4 [50]. Furthermore, it is feasible to assume that DNA ligases other than LIG4 are overexpressed in GBM cell lines and thereby compete with LIG4 for inhibitor binding [50]. Thus, we recommend the re-evaluation of LIG4 as a candidate for targeted radiosensitization of GBM as soon as inhibitors with improved pharmacological profile are available in order to clarify if LIG4 is mechanistically involved in GBM radioresistance or if the correlation of its expression levels with inherent radioresistance has no causal implications.

### Functional validation reveals ATM inhibition as a strategy to increase the sensitivity of GBM cells to TMZ treatment to be complex

The unexpected strong correlation between inherent resistance of GBM cells to TMZ and mRNA expression levels of ATM prompted us to investigate whether inhibition of ATM could indeed increase the efficacy of TMZ treatment in resistant GBM cells. To this end, we employed the ATM inhibitor KU-60019 [41]. Inhibitor treatment of GBM cells with different MGMT promoter methylation status and mRNA/protein expression levels (Fig. 1) was performed 1 h prior to the addition of TMZ for 24 h. Then, medium was replaced, and colony formation was allowed in inhibitor-free medium (Fig. 4C). KU-60019 treatment had no obvious effects on TMZ sensitivity of GBM cells negative (U138-MG) or moderately positive (U251-MG) for MGMT promoter methylation, and accordingly positive for MGMT mRNA expression (Fig. 1)—even though the expression levels in these cell lines were lower than in normal astrocytes (Fig. 1A). Nevertheless, slight but statistically not significant sensitizing effects towards TMZ by KU-60019 were observed for A172 cells (Fig. 4C), a cell line positive for MGMT promoter methylation and very low MGMT mRNA and protein expression levels (Fig. 1). Similar findings for an involvement of ATM in TMZ resistance of MGMT low expressing GBM cells have been also reported by others [51–53]. Thus, it needs to be further investigated if ATM inhibition may represent a sensitization approach for TMZ treatment of GBM with low MGMT expression.

Collectively, our results show that correlating clonogenic survival data with mRNA expression data followed by functional validation in form of pharmacological perturbation represents a feasible approach to identify markers and potential vulnerabilities of radio/chemotherapy resistance and that the major targets identified by this approach deserve further characterization.

## Discussion

Here, we describe a systematic, integrative approach for the analysis of inherent treatment resistance of GBM cells. It is based upon the integration of clonogenic survival data with mRNA expression data and aims at the identification of regulators involved in inherent treatment resistance which represent interesting targets for sensitizing strategies in combined modality radio/chemotherapy. We chose a cell line panel of GBM as a model of an inherently highly treatment-resistant cancer entity [2] and the DNA damage response (DDR) as a compilation of genes with high relevance for resistance against radiotherapy and/or DNA targeting chemotherapy [54].

Our correlative analyses confirmed markers of GBM treatment resistance that are already well-known, such as MGMT expression in the context of TMZ resistance. It also disclosed potential targets for pharmacological sensitization that have previously been validated, such as PARP1 and HSP90 with regard to resistance against radiotherapy [18, 37–39, 55]. These findings serve as proof-of-concept for our strategy. Furthermore, our screen identified additional, so far underestimated candidates for targeted sensitization, including ATR in the context of GBM radioresistance. Since functional validation of ATR's involvement in GBM radioresistance with the ATR inhibitor AZD-6738 provided very convincing radiosensitization of GBM cells in vitro, and similar evidence has also been provided by others [46], ATR inhibition in combined modality GBM radiotherapy certainly deserves more in-depth preclinical investigation. Similarly, the association of ATM expression with TMZ resistance in the context of MGMT low expressing GBM is an interesting novel finding disclosed by our study. Since similar findings were published before [51–53], and ATM inhibitors are currently undergoing clinical trialing in GBM (NCT03423628, NCT05182905), this observation should be further investigated in future. Nevertheless, there were also genes whose expression levels correlated with therapy resistance and whose functional involvement could not be validated. As such, inhibition of LIG4 did not noticeably sensitize GBM cells towards radiotherapy. This may derive from the correlative, not causative nature of our screen [56] as well as from the suboptimal pharmacological characteristics of the used inhibitor L189 [50]. So, the performance of LIG4 targeting in combined modality GBM radiotherapy should be preclinically re-evaluated as soon as improved next generation LIG4 inhibitors are available.

Overall, our findings demonstrate the feasibility as well as the limitations of this correlative approach for the identification of markers of inherent radio/chemoresistance and potential targets for pharmacological sensitization. This is not restricted to glioblastoma, and instead may be adapted to other cancer entities as well [19]. It is also not limited to targeted expression analyses of small gene sets, such as the DDR selected for our study. In fact, we consider it to be well-applicable to larger gene sets, whole transcriptome data, and other OMICs levels. Very recently, we also applied it to functional datasets of cell fate decisions and identified the early induction of tumor cell senescence in conjunction with the production of senescence-associated cytokines as therapeutically targetable determinants of radioresistance in head-and-neck squamous cell carcinoma (HNSCC) [20].

## Conclusions

The persistent limitation in treatment success of radio/chemotherapy in highly resistant cancer entities such as GBM demands for the identification of novel markers that contribute to therapy resistance and represent targets for pharmacological interference. Here, we present a systematic, integrative approach correlating clonogenic survival data with mRNA expression data to identify markers and drivers of radio/chemotherapy resistance in GBM. Our findings provide proof-of-concept for the feasibility of this approach as well as its limitations and open the perspective to apply it to other cancer entities and large-scale molecular data sets.

## Data Availability

The data presented in this study are available from the corresponding author upon reasonable request.

## Abbreviations

53BP1: P53 binding protein 1
ALAS: 5’-Aminolevulinate synthase-1
ATM: Ataxia telangiectasia mutated
ATR: Ataxia telangiectasia and RAD3 related
ATRX: Alpha thalassemia/mental retardation syndrome X-linked
B2M: β2-Microglobulin
BLM: Bloom syndrome RecQ like helicase
BSA: Bovine serum albumin
CDKN1A: Cyclin-dependent kinase inhibitor 1A
DDR: DNA damage response
DMEM: Dulbecco’s modified eagle medium
DMSO: Dimethylsulfoxide
EDTA: Ethylene diamine tetraacetic acid
EXO1: Exonuclease 1
FCS: Fetal calf serum
FEN1: Flap-structure specific endonuclease 1
γH2AX: Phosphorylated histone variant H2AX
GBM: Glioblastoma
HNSCC: Head-and-neck squamous cell carcinoma
HSP90AA1: Heat shock protein 90 AA1
HSP90AB1: Heat shock protein 90 AB1
IR: Ionizing radiation
LIG4: DNA Ligase 4
MGMT: O^6^-Methylguanine-DNA-methyltransferase
MRE11A: Meiotic recombination 11 homolog A
MRN: MRE11-RAD50-NBN
NBN: Nibrin
NHEJ: Non-homologous end-joining
PBS: Phosphate-buffered saline
PC1: Principal component 1
PCA: Principal component analysis
qRT-PCR: Quantitative realtime polymerase chain reaction
RAD50: RAD50 double strand break repair protein
RPA1: Replication protein A1
STR: Short tandem repeat
TMZ: Temozolomide
TP53: Tumor-suppressor protein 53
XRCC: X-ray repair cross-complementing
